# Medical History from the Earliest Times

**Published:** 1893-07-01

**Authors:** E. T. Withington


### Medical History from the Earliest Times.

By E. T. Withington, M.A., M.B. Oxon.

### LX.—THE VITALISTS.

In order to bring together two closely allied medical systems, we must deviate a little from the strictly chronological order, for Paul Joseph Barthez, the foremost exponent of what may be called pure vitalism, was born in the year Stahl died (1734). Though so similar in doctrine, he presents a striking contrast to his predecessor in everything else. Stahl is the most obscure, Barthez one of the clearest of medical writers. Stahl was a metaphysician; Barthez, though he represents a metaphysical system, was a man of a decidedly practical and scientific mind. The famous Chancellor of Montpellier lived during the period of the so-called Anglomania in France, and he prided himself upon being a disciple of the great English philosophers—Bacon, Locke, and Newton. Science, he declares, has nothing to do with the essence of things, but is the study of phenomena, and medical science studies the phenomena presented by the human body in health and disease. Some of these have their origin in the mind or will, as, for instance, the various voluntary movements. Others, such as the action of bones and joints, obey the laws of mechanics. But between these comes a third and most important class of phenomena which especially concern the physician, and which are not to be explained either by mind or mechanism. Not by mind, for we are unconscious of them; and if Stahl urges that this is due to habit, through which we also become unconscious of the voluntary acts involved in speaking, walking, or playing an instrument, Barthez replies that we can always regain this consciousness by a slight effort of attention, but no exertion of will can give us control over the circulation, digestion, or any other vital function. Moreover, the vital activities are the same in all men, but their souls or minds differ widely; a vast number of vital functions are carried on at the same time with perfect regularity, but it is characteristic of the mind that it can only give full attention to one thing at once. Against the theory that life is the result of structure or organisation, Barthez argues that a mechanism however complex is in itself absolutely passive. He points out that life may be destroyed without any apparent lesion of structure, while it may survive the most severe injuries, and he remarks that if vitality depends upon a definite arrangement of particles, the fluids of the body, in which there can be no such definite arrangement, must be dead; yet there is strong evidence that the blood, if not the life itself, at any rate possesses vital properties.

Having by these and other arguments shown the weakness of opposing theories, Barthez concludes that we must assume the existence of something which is neither soul nor body, and which he calls "vital principle." Whether this vital principle is a mere property or an independent being he does not know; he compares it, in fact, to the "X" or unknown quantity of the algebraist; but he evidently leans strongly towards the latter alternative, for in discussing its final fate he supposes that it may either die with the body, become reincarnate in some other body, or be absorbed into some universal vital principle.

The Barthezian or vitalistic pathology need not detain us, for it is simply that of Stahl and Van H-lmont with a different set of names. Disease is the effort of the vital principle to resist some harmful agency, or it is due to a morbid idea "manifesting itself by alterations in sensibility, abnormal movements, or an aberration in those acts which regulate the chemical constitution of the humours," or, finally, it may be caused by excess or defect of general vital activity. But it is characteristic of Barthez that he allows his theory to have very little influence upon his practice, and his therapeutic doctrines present several interesting and original features. There are, he says, three ways of treating disease—a natural, an analytical, and an empiric. The first bids us assist Nature (or the vital principle) in her efforts, as for instance by giving an emetic in nausea, or a purgative in some forms of diarrhœa; and the cautious physician will always employ this mode of treatment in cases where the termination of the disease is naturally favourable. But it is on the analytic method that Berthez lays greatest stress. He observes that the same affection is often benefited by different drugs, and that the same remedy is useful in many forms of illness, and he concludes from this that most disorders are compounded of several elementary affections. In such cases it should be the physician's object to distinguish these, and to attack them separately "by means proportionate to their force and influence." Thirdly, we may fall back upon the empiric method, of which there are three varieties: (1) A "methode perturbatrice," which attempts to dissipate an existing chronic affection by substituting a more acute one, "as Sydenham, and Boerhaave after him, were wont to cure obstinate agues by exciting diaphoresis or purgation a little before the attack"; (2) a "methode imitative," by which the vital principle is directed into the path by which Nature usually cures similar diseases; and (3) the employment of remedies which have been found to have a specific relation to the disease in question, or, in other words, which seem to cure the disorder without any intermediate action.

Though Barthez was far the ablest of the pure Vitalists, the want of any close connection between his theory and practice makes him a somewhat imperfect representative. He repeatedly protests that he does not mean to explain anything by his "vital principle," which is simply a short way of expressing his belief that life is not the result of either bodily or mental action; and he points out that Newton had similarly used the abstract term "gravitation" as a short way of saying that all material particles tend to approach one another with a definite acceleration. But his followers not unreasonably asked what was the use of assuming a vital principle at all if it explained nothing. Just as the successors of Newton converted "gravitation" into an actual force inherent in matter, and directing its movements, so the adherents of Barthez exalted his "vital principle" into a spiritual being which resides in the body and endows it with life. They thus approached still more closely to the Animists, for though it may be of great import philosophically, whether we suppose that life is the soul or some other spiritual entity, the results from the view of practical medicine are nearly identical. A mind diseased or a disordered vital principle seem to be equally beyond the reach of merely material drugs, and both Animists and Vitalists thus tended either to reduce their therapeutics within the narrowest limits, as was the case with Stahl, or to assume that medicines contain peculiar semi-spiritual or dynamic powers enabling them to come into direct contact with the immaterial source of life and disease.

The most striking example of this latter tendency is seen in the doctrine of Hahnemann, whose system may be considered historically as a union of the extreme vitalistic theories with the mysticism of the sixteenth century. Paracelsus tells us that every substance contains a hidden quintessence or spirit of life. In man and animals this spirit cannot be isolated, for they die in the process. "Wherefore no quintessence can be got from flesh and blood. If we could draw out the life of the heart without destroying it, then we could maintain our lives for ever without disease by means of this quintessence." But the spirits of plants and minerals may be separated by the art of the alchemist, and these form the Paracelsic arcana or specific medicines. Similarly Hahnemann holds that all drugs contain "a dynamic spiritual power," which is awakened and brought to life not by chemistry, but by "dynamisation," "a process unknown before my time," and consisting in rubbing the dry substances in a mortar, and shaking the fluids, "which is a kind of rubbing." This is not a mere attenuation, though repeated attenuations are necessary "that the rubbing or shaking may penetrate more fully into the essence of the drug and so set free its more deeply-seated medical powers, which can then act upon our life in an almost spiritual manner" and so cure diseases, for "diseases are solely spiritual derangements of the spiritual vital force which animates the human body."

But we are already far beyond the general course of this history, and must now go back to consider the most important of the eighteenth century sects—the Organicists.

